# Mapping the neural substrate of high dual-task gait cost in older adults across the cognitive spectrum

**DOI:** 10.1007/s00429-024-02873-6

**Published:** 2025-01-04

**Authors:** Pauline Ali, Mickaël Dinomais, Matthieu Labriffe, Frederico Pieruccini-Faria, Manuel Montero-Odasso, Robert Bartha, Cédric Annweiler

**Affiliations:** 1 Department of Medical Biophysics, Schulich School of Medicine & Dentistry, Western University, 1151 Richmond Street, North London, ON N6A 5C1 Canada; 2 Laboratoire Angevin de Recherche en Ingénierie des Systèmes, Centre de Rééducation et Réadaptation des Capucins, University of Angers, 62 avenue Notre Dame du Lac, EA731549000 Angers, Pays de la Loire, France; 3 Department of Physical and Rehabilitation Medicine, Angers University Hospital, University of Angers, 4 rue Larrey, 49000 Angers, France; 4 Department of Radiology, Angers University Hospital, 4 rue Larrey, 49000 Angers, France; 5 Gait and Brain Lab, Parkwood Institute, Lawson Health Research Institute, 550 Wellington Road South, London, ON N6C 0A7 Canada; 6 Department of Medicine, Division of Geriatric Medicine, Schulich School of Medicine & Dentistry, Western University, 550 Wellington Road South, London, ON N6C 0A7 Canada; 7 Department of Epidemiology and Biostatistics, Schulich School of Medicine & Dentistry, Western University, London, 550 Wellington Road South, London, ON N6C 0A7 Canada; 8 Center for Functional and Metabolic Mapping, Robarts Research Institute, Schulich School of Medicine & Dentistry, Western University, 100 Perth Drive, London, ON N6A 5K8 Canada; 9 UNIV ANGERS, UPRES EA 4638, University of Angers, Angers, France; 10 Department of Geriatric Medicine and Rehabilitation, Research Center On Autonomy and Longevity, University Hospital, 4 Rue Larrey, 49000 Angers, France

**Keywords:** Dual-task cost of gait, Gray matter volume, Neural substrate, Cognitive impairment, Aging

## Abstract

The dual task cost of gait (DTC) is an accessible and cost-effective test that can help identify individuals with cognitive decline and dementia. However, its neural substrate has not been widely described. This study aims to investigate the neural substrate of the high DTC in older adults across the spectrum of cognitive decline. A total of 336 individuals from the GAIT study cohort were analyzed, including cognitively healthy (N = 122, 71 ± 3.6 years), those with mild cognitive impairment (N = 168, 71 ± 5.3 years), and those with dementia (N = 46, 80 ± 5.7 years). A DTC of 20% or greater was considered to indicate a high level of slowing down while performing successively two verbal tasks (counting backwards task by ones and naming animals). Voxel-based morphometry was employed to investigate differences in gray matter volume (GMV) between groups, which were dichotomized according to the DTC. A high DTC in the whole population (N = 336) was associated with a smaller GMV in the bilateral temporal lobe across both dual-task conditions. A moderation analysis was employed to compare the neural substrate between cognitive status groups. This revealed that the dementia group exhibited an additional cluster located in the left precentral gyrus with GMV loss associated with a high naming animals DTC, in contrast to the other cognitive groups. These results provide new evidence on why dual-task gait capabilities deteriorate in normal and pathological cognitive aging. A more precise understanding of the neural substrate associated with high DTC and cognitive status would help elucidate its use in clinical and research settings.

**Supplementary Information:**

The online version contains supplementary material available at 10.1007/s00429-024-02873-6.

## Introduction

The dual-task gait test is a rapid and cost-effective method for assessing the cognitive capabilities of older adults during walking. It can be employed in both clinical and research settings and provides insight into how cognitive resources are used in more natural real-life conditions (Plummer et al. 2015; Montero-Odasso et al. 2019). Previous studies have demonstrated that impairment in specific cognitive domains, such as attention, executive function, and working memory, is associated with slower dual-task gait speed (Montero-Odasso et al. 2009a, b). This slowdown in gait or the dual-task gait cost (DTC), while performing a concurrent mental task, is frequently higher in individuals with mild cognitive impairment (MCI) and dementia (Cullen et al. 2019; Muir et al. 2012). Moreover, a slowing of gait speed by more than 20% when performing a dual task compared to a single task (Sakurai et al. 2019; Montero-Odasso et al. 2017) is associated with faster progression to dementia in MCI (Montero-Odasso et al. 2017). Therefore, the high DTC has great potential as a clinical marker of dementia risk. The DTC involves cerebral systems that control both cognitive and gait functions, which may share a common neural substrate (Yogev-Seligmann et al. 2008; Al-Yahya et al. 2011).

Despite the growing body of literature supporting the use of DTC, only a few neuroimaging studies have focused on its neural basis with several methodological limitations including sample size, clinical characterization, few regions of interest and neuroimaging metrics extracted (Sakurai et al. 2019; Subotic et al. 2023; Tripathi et al. 2019; Hupfeld et al. 2022). Nevertheless, it is necessary to investigate the underlying pathophysiological processes associated with this parameter and identify the brain areas involved in DTC abnormalities to better understand their full potential for the diagnosis of dementia (Tian et al. 2023). Previous literature on brain structural analysis and dual-task gait performance has identified an association between regional gray matter volume (GMV) loss and poorer gait, predominantly in the frontal and temporal lobes, the lateral occipital cortex, and the postcentral gyrus. These findings suggest a potential role for these areas in the central control of the cognitive-motor interaction (Allali et al. 2019; Tripathi et al. 2019; Sakurai et al. 2019; Doi et al. 2017). These studies included cohorts of older adults with various stages of cognitive level (from healthy to mild cognitive impairment) without directly questioning the impact of these cognitive statuses on the neural substrate of the dual-task performance (Koppelmans et al. 2022). The observed high DTC in individuals with dementia may be caused by the alteration of additional functions necessary to achieve a walk and a cognitive task simultaneously. It is also known that individuals with cognitive decline, have a loss of GMV in several vulnerable regions frequently found abnormally smaller in individuals with dementia. For instance, the most prevalent form of dementia, Alzheimer’s Disease (AD), is characterized by a significant reduction in GMV in the parietal and cingulate cortices, even before the typical clinical manifestations (e.g. memory impairment). In less advanced stages including amnestic MCI (aMCI) (Karas et al. 2004) a significant reduction in GMV in the medial temporal lobe in comparison to healthy cognitively normal individuals can be identified (Karas et al. 2004; Pennanen et al. 2005). This GMV loss has been correlated with neuropsychological performance in patient with aMCI and AD (Arlt et al. 2013). Importantly, the aforementioned vulnerable structures could be also involved in dual-task performance (Subotic et al. 2023; Doi et al. 2017). Conversely, the neural mechanisms underlying gait dysfunction in neurodegenerative disorders remain poorly understood, as evidenced by the literature review conducted by Koppelmans et al (2022). It is, therefore, of great interest to ascertain whether localized GMV loss in dementia and preclinical stages are involved in poorer dual-task gait performance. The dual-task gait performance, expressed in DTC, is of particular interest as it is more closely related to cognitive processing during gait, than either gait or cognitive measures alone (Montero-Odasso et al. 2019). To the best of our knowledge, no studies to date have systematically and comprehensively investigated the neural basis of high DTC across the cognitive spectrum.

The objective of the present study was to determine whether there is a neural substrate of high DTC in community-dwelling older adults across the cognitive spectrum and, if such a substrate exists, whether it varies depending on the cognitive status of individuals.

## Methods

### Population

The participants were recruited from the “Gait and Alzheimer Interactions Tracking” (GAIT) cohort, which conducted a cross-sectional study between November 2009 and November 2015 to compare the gait characteristics of individuals with varying cognitive profiles, ranging from healthy individuals to those with dementia. The study procedure has been described in detail previously (Beauchet et al. 2019). Participants had to be 60 years of age or older, ambulatory, community-dwelling, with adequate understanding of French language, and without acute medical illnesses in the past month. Additionally, they were required to have a MMSE score of at least > 10, visual acuity of at least ≥ 2/10, no severe depressive symptoms (15-item Geriatric Depression Scale score ≤ 10), and no acute medical illnesses in the past month. Individuals were excluded from participation if they had preexisting locomotor disorders, a history of stroke or sensorimotor sequelae from the central nervous system, any acute medical or surgical condition less than 3 months old, a score of greater than 10 on the 15-item Geriatric Depression Scale, or if they were unable to walk unaided for less than 15 min. For this analysis, we only included participants who underwent a brain MRI and a dual-task gait assessment. Participants were included after providing written informed consent to participate in the study. The research protocol was approved by the Angers Ethics Committee (CPP Ouest II-2009–12) and registered as ClinicalTrials.gov n° NCT01315717.

### Clinical assessment

#### Cognitive assessment

The Memory clinic of Angers University Hospital (France) assessed all participants. Cognitive status diagnoses were made during multidisciplinary assessments involving geriatricians, neurologists, and neuropsychologists. Controls were subjects with normal neuropsychological results (MMSE score > 25 and negative Winblad criteria) (Winblad et al. 2004). Dementia was diagnosed using the Diagnostic and Statistical Manual of Mental Disorders (Fourth Edition, Text Revision) (Quinn 1999) and NINCDS/ADRDA criteria (Dubois et al. 2007). MCI was diagnosed according to the criteria detailed by Winblad et al (2004). These criteria require that the person is neither normal nor with dementia, there is evidence of cognitive deterioration shown by either objectively measured decline over time and/or subjective report of decline by self and/or informant in conjunction with objective cognitive deficits, and activities of daily living are preserved, and complex instrumental functions are either intact or minimally impaired.

Participants underwent evaluation using a neuropsychological battery that included standardized assessments of various cognitive domains. Global cognition was assessed using the Mini-Mental State Examination (MMSE), (Folstein et al. 1975) Alzheimer's Disease Assessment Scale-Cognitive Subscale (ADAS-Cog), (Kueper et al. 2018) and Frontal Assessment Battery (FAB) (Dubois et al. 2000). To assess instrumental functions, language abilities were evaluated with the ADAS-Cog and specific subtests from the Wechsler Adult Intelligence Scale-Revised (WAIS-R). Praxes were examined through the assessment of five different upper limb postures, along with related items from the ADAS-Cog. Visuo-spatial abilities were assessed using the Visual Object and Space Perception (VOSP) test battery as described by Warrington and James (1991). Memory performance was evaluated through a French version of the Free and Cued Selective Reminding Test (Dion et al. (2015), in addition to the Digit Span task (both forward and backward) derived from the WAIS-R. To assess executive functions, the Trail Making Test (TMT) parts A and B was administered.

#### Gait evaluation

Gait assessment was conducted using a Gaitrite© System, which is 972 cm long, to evaluate spatiotemporal gait parameters in older adults according to the European guidelines (European GAITRite® Network Group, Kressig, et Beauchet 2006). Participants completed three gait trials in random order to minimize the effects of learning and fatigue. They were instructed to walk at their usual gait speed (cm/s) while wearing their own footwear. During the dual-task trials, participants were instructed to walk at their usual pace without prioritizing either the gait or cognitive task. They were asked to perform the following cognitive tasks aloud: (i) counting backwards (CB) from fifty to zero one by one and (ii) naming animals (NA). The dual-task gait cost (DTC) was calculated for each trial using the appropriate gait speed with the formula: DTC = [(single-task gait speed—dual-task gait speed)/single-task gait speed] × 100 (Plummer et al. 2015). DTC was expressed as a percentage of slowing from the usual gait speed due to the added cognitive task. High DTC was defined as a slowing down of 20% or more, while low DTC is defined as less than 20%, as determined by our previous work (Sakurai et al. 2019; Montero-Odasso et al. 2017). The decision to utilize dichotomous DTC instead of continuous DTC was predicated on the observation that the 20% cutoff point has been demonstrated to be more clinically relevant and sensitive, given its capacity to account for intersubject variability (Montero-Odasso et al. 2017). The reliability of this protocol has been previously demonstrated (Montero-Odasso et al. 2009a, b).

#### MRI acquisition

Brain imaging was performed using either a 1.5-Tesla MRI scanner (until 2011, Magnetom Avanto; Siemens, Erlangen, Germany) or a 3-Tesla (from 2011, Magnetom Skyra, Siemens, Erlangen, Germany) using a standard MRI protocol including 3D T1-weighted magnetization prepared rapid acquisition gradient echo (MP-RAGE) images (for 1.5-Tesla MRI: acquisition matrix = 256 × 256 × 144, field of view (FOV) = 240 × 240 × 187 mm, echo time (TE)/repetition time (TR)/inversion time (TI) = 40.07 ms/2170 ms/1100 ms); for 3-Tesla MRI acquisition matrix = 256 × 256x128, FOV = 240 × 240x176 mm TE/TR/TI = 2.98 ms/2300 ms/900 ms).

#### MRI processing

The analysis utilized the CAT12 toolbox (Computational Anatomy Toolbox 12, vCAT12.8.2), an extension of SPM12 (Statistical Parametric Mapping, v7771), to conduct the voxel-based morphometry (VBM) on anatomical T1-weighted images. These images underwent automated segmentation into gray matter, white matter, and cerebrospinal fluid. Additionally, the volume of white matter hyperintensities (WMHs) was delineated as a separate class using the “expert mode” (Gaser et al. 2022). The pre-processing steps, adhering to the standard VBM protocol, involved comprehensive brain tissue segmentation and spatial alignment using the Shooting template (Ashburner et al. 2000, 2011; Gaser et al. 2022). The resulting gray matter volume (GMV) images, aligned to the template, were of a resolution of 1.5 × 1.5x1.5. Quality assurance was robust, involving both automated and visual inspections to ensure the absence of artifacts. To minimize individual gyral variations, the data were smoothed using a 6 mm full width at half maximum (FWHM) Gaussian Kernel. An absolute gray matter threshold of 0.1 was applied to ensure the inclusion of solely gray matter regions in the statistical analyses.

To extract GMV data from each significant cluster, binary masks were applied to the non-smoothed, modulated GMV images of each participant.

#### Statistics

To describe the study population based on cognitive status groups (control, individuals with MCI, and those with dementia), we utilized appropriate statistical tests such ANOVA with post-hoc test and Tukey correction, or Chi-square test as appropriate. To adjust for skewness, we log-transformed WMHs volumes. We conducted statistical comparisons using JASP (Version.17.1, https://jasp-stats.org/).

To compare GMV (outcome) between the high and low DTC groups (predictor), we used the general linear model approach implemented in SPM12. Statistical analysis was conducted using a whole-brain ANOVA that included covariates such as age, sex, educational level (post-secondary or none), MRI field strength (1.5 T or 3 T), WMHs, and total intracranial volume (TIV). The VBM results were adjusted for multiple comparisons using the family-wise error (FWE) method, with significance set at *p* < 0.05. This correction was implemented alongside threshold-free cluster enhancement (TFCE) with 5000 permutations (Spisák et al. 2019). Significant clusters were identified on VBM statistical maps. These clusters were defined as having notable differences in gray matter (negative or positive) and comprising at least 10 contiguous voxels. The locations of these clusters were determined using the Automated Anatomical Labeling version 3 (AAL3) atlas (Rolls et al. 2020).

To investigate the effect of cognitive status on the relationship between GMV (outcome) and high versus low DTC (predictor), a moderation analysis was conducted using the PROCESS macro on R Core Team (Version 4.2.3, 2023, https://www.R-project.org/) and following Hayes’ guidelines. The PROCESS macro provides results that include logistic regression and the interaction with the regression coefficient if the interaction is significant.

**Fig. 1:**
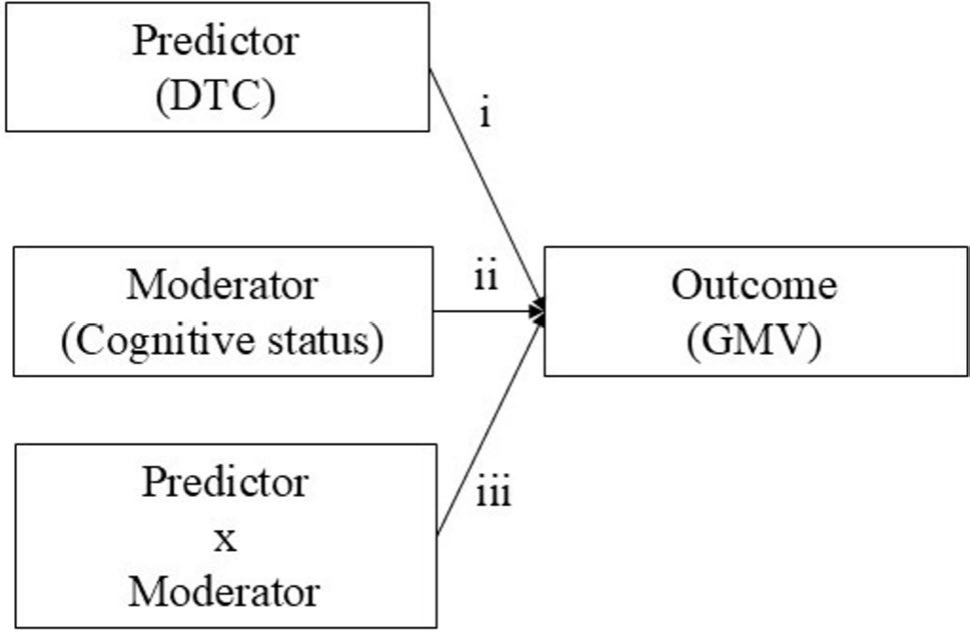
Output model of Cognitive Status Moderation effect on the relationship between Gray Matter Volume (GMV) and Dual-Task Cost (DTC)

To probe for the moderation effect of the cognitive status (H1), it was necessary for the relationships for (i), (ii), and (iii) to be significant (Fig. 1). These relationships include (i) the direct effect of the predictor (DTC) on GMV, (ii) the direct effect of the moderator (cognitive status) on GMV, and (iii) the direct interaction effect (DTC x cognitive status) on GMV. In R PROCESS, the software automatically calculates the interaction effect and produces the likelihood ratio test explained by the moderating effect of cognitive status (increase due to interaction). The potential moderators, each assessed within an independent moderation model, were as follows: dementia versus control groups, MCI versus control groups, dementia versus MCI groups. A *p* < 0.05 was considered statistically significant. The moderation model was adjusted for covariates including age, sex, educational level, TIV, and MMSE.

## Results

A total of 336 participants were included in the current analysis, consisting of 122 control participants, 168 participants with MCI, and 46 participants with dementia. 3 participants (1 healthy control and 2 people with dementia) did not perform the NA gait task.

### Cohort characteristics

Table 1 summarizes the main demographic characteristics. The dementia group was significantly older (79 ± 5.7 years) than the control (71 ± 3.6, *p* < 0.001) and MCI (73 ± 5.3, *p* < 0.001) groups and the MCI group was older than the control group (*p* = *0.0*2). Additionally, the dementia group had a higher proportion of women compared to the control group (*p* < 0.001). The MCI group had a higher body mass index (27 ± 4) than the control group (25 ± 3, *p* = 0.02). There were no differences in comorbidities between the groups.

**Table 1:** Demographic Characteristics of the Participants According to Cognitive Status:

Total population (n = 336)	Control Group (n = 122)	MCI (n = 168)	Dementia (n = 46)	*p* value	Post hoc analysis		
					Control versus MCI	Control versus Dementia	MCI versus Dementia
Age (years), (mean ± SD)	71 ± 3.6	73 ± 5.3	79 ± 5.7	** < 0.001**	**0.02**	** < 0.001**	** < 0.001**
Women, n (%)	57 (47%)	60 (36%)	31 (66%)	** < 0.001**	0.18	0.07	** < 0.001**
Post secondary education, n (%)	80 (66%)	94 (56%)	15 (32%)	** < 0.001**	0.10	** < 0.001**	**0.003**
Body mass index (kg/cm^2^) (mean ± SD)	25 ± 3	27 ± 4	26 ± 4	**0.03**	**0.02**	0.75	0.46
Number of comorbidities (mean ± SD)	2 ± 1.5	2 ± 1.7	3 ± 2.3	0.14	0.36	0.15	0.60
Cognitive Profile n (%)	NA	Amnestic: 40 (24%) Nonamnestic: 110 (65%) Multidomain: 18 (11%)	AD*: 37 (80%) Mixed: 9 (20%)	NA	NA	NA	NA
Total intracranial volume (mL) adjusted on sex, (mean ± SD)	1480 ± 132	1524 ± 139	1475 ± 137	0.10	0.12	0.27	0.98
Volume of white matter hyperintensities (mL)	3.1 ± 30.0	5.5 ± 7.2	12.2 ± 15.9	** < 0.001**	** < 0.001**	** < 0.001**	** < 0.001**
	Global cognition						
MMSE score † (mean ± SD)	28 ± 1.3	27 ± 1.9	24 ± 4.5	** < 0.001**	** < 0.001**	** < 0.001**	** < 0.001**
ADAS-Cog score ‡ (mean ± SD)	4.1 ± 1.5	5.9 ± 2.1	15.4 ± 6.6	** < 0.001**	** < 0.001**	** < 0.001**	** < 0.001**
	Gait speed						
Single gait (cm/s), (mean ± SD)	112 ± 19	106 ± 19	80 ± 23	** < 0.001**	**0.031**	** < 0.001**	** < 0.001**
Counting backward (cm/s), (mean ± SD)	114 ± 23	103 ± 24	75 ± 27	** < 0.001**	** < 0.001**	** < 0.001**	** < 0.001**
Naming animals (cm/s), (mean ± SD)	100 ± 25	87 ± 27	61 ± 25	** < 0.001**	** < 0.001**	** < 0.001**	** < 0.001**
	High dual-task cost (≥ 20%)						
Counting Backward	6 (5%)	26 (15%)	10 (22%)	**0.003**	**0.02**	**0.01**	0.77
Naming animals	34 (28%)	74 (44%)	26 (59%)	** < 0.001**	**0.02**	**0.001**	.21

Where appropriate the mean is shown with standard deviation in parentheses

Comparison based on one-way ANOVA and post hoc test with Tukey correction or Chi-square test as appropriate

*P*-value significant (i.e., < 0.05) in bold

1individual in the control group and 2 participants with dementia didn’t perform the naming animals task

*SD* standard deviation, *n* number of participants

*AD = Alzheimer’s Disease, Mixed = mixed form of dementia

^†^ MMSE = Folstein Mini-mental State Examination (scores range from 0 to 30, higher scores representing better function),

^‡^ ADAS-Cog = Alzheimer’s Disease Assessment Scale (score ranges from 0 to 70 with higher scores suggesting greater impairment, 58 missing data),

Among the participants in the MCI group, 40 individuals (24%) exhibited amnestic (aMCI) features, while 110 individuals (65%) displayed non-amnestic (naMCI) symptoms. Additionally, 18 individuals (11%) showed impairments across multiple cognitive domains. Of those diagnosed with dementia, 37 individuals (80%) were diagnosed with Alzheimer’s Disease (AD), while 9 individuals (20%) had a mixed form of dementia involving vascular and other neurodegenerative factors, excluding AD.

After adjusting for sex, no significant differences in TIV were found among the three groups (*p* = 0.10, Table 1). Participants with dementia had a higher volume of WMHs than the control participants (12.2 ± 15.9 mL vs. 3.1 ± 30.0 mL, *p* < 0.001, Table 1) and people with MCI (5.5 ± 7.3 mL, *p* < 0.001, Table 1), and people with MCI had a higher volume of WMHs than the control group (*p* < 0.001, Table 1).

As expected, the MMSE and ADAS-Cog scores were significantly lower in the dementia group compared to the control and MCI groups (*p* < 0.001, Table 1). Participants with a cognitive impairment (including those with MCI and dementia status) exhibit significantly lower dual-task gait speed and higher DTC (indicating worse performance) across all conditions (Table 1). There was no significant difference of DTC between individual with MCI and those with dementia (unadjusted and adjusted from age, sex, educational level and MMSE).

A comparison of the high and low DTC groups revealed that those with high CB DTC exhibited a lower MMSE score than those with low CB DTC (*p* = 0.032). Nevertheless, no significant differences in WMHs were observed between the two groups based on CB DTC load (*p* = 0.814) (eTable1). In the NA DTC groups, individuals with high NA DTC had significantly lower TIV volume (*p* = 0.047) and a higher volume of WMHs (*p* = 0.02). Additionally, a trend towards a lower MMSE score was observed in the high NA DTC group, though this was not statistically significant (*p* = 0.051) (eTable 1). Of the 42 participants who demonstrated a high CB DTC, 40 also exhibited a high NA DTC. The remaining two individuals who exhibited a high NA DTC but a low CB DTC were classified as follows: one with naMCI and one with probable AD.

### Brain gray matter volume (GMV) difference following the load of dual task cost (DTC)

**Fig. 2:**
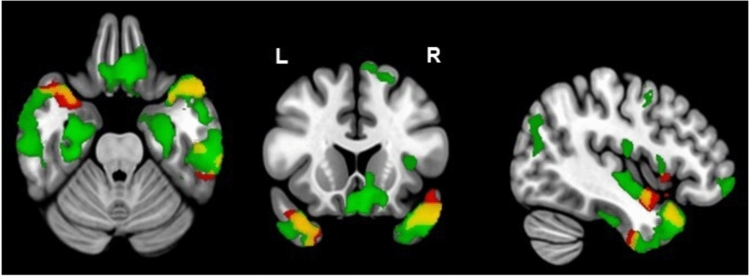
Mapping of Gray Matter Volume (GMV) Difference Between High versus Low Dual-Task Cost (DTC): (Counting backwards (CB) is shown in red and Naming Animals (NA) in green) in all three orientations. The red cluster shows a significant loss of GMV in individuals with high CB DTC in comparison to low CB DTC. The green cluster shows a significant loss of GMV in individuals with high NA DTC compared to low NA DTC. The yellow area shows the overlap of the both dual-task cost conditions. Results are showed adjusted on age, sex, educational level, MRI type, WMHs, and TIV with a significance of *p* < *0.0*5 corrected for multiple comparisons

Among the entire population, individuals with a higher counting backward dual-task cost (CB DTC) exhibited a smaller GMV compared to those with a low CB DTC. This was observed in five clusters located in the left and right temporal lobes (1.2 ± 0.2 vs. 1.1 ± 0.2 mL for the cluster 1, see Table 2 and Fig. 2).

**Table 2:** Gray Matter Volume Difference Between High versus Low Dual-Task Cost (n = 336 for CB DTC and n = 333 for NA DTC)

Cluster No.	Cluster Size (no. of voxel)	Brain Region (cluster peak)	GMV (mL)		MNI Coordinates of the maximum intensity voxel			TFCE-value	*p-FWE-c*
			CB DTC < 20%	CB DTC ≥ 20%					
			(n = 294)	(n = 42)					
1	1723	Left Middle Temporal	1.2 ± 0.2	1.1 ± 0.2	− 45	− 2	− 18	1123	**0.01**
2	3091	Right Temporal Pole	1.5 ± 0.2	1.4 ± 0.2	36	17	− 33	1093	**0.01**
3	412	Left Middle Temporal	1.2 ± 0.2	1.1 ± 0.2	− 66	− 15	− 14	891	**0.03**
4	425	Left Inferior Temporal	1.6 ± 0.3	1.5 ± 0.2	− 42	− 8	− 39	890	**0.03**
5	101	Left Inferior Temporal	1.5 ± 0.2	1.4 ± 0.2	− 54	− 42	− 21	807	**0.049**

			**NA DTC < 20%**	**NA DTC ≥ 20%**					
			**(n = 199)**	**(n = 134)**					
1	35,883	Left Lingual	1.2 ± 0.2	1.1 ± 0.2	− 2	− 72	− 5	2951	** < 0.001**
2	8550	Right Inferior Temporal	1.5 ± 0.2	1.4 ± 0.2	60	− 21	− 26	2291	** < 0.001**
3	756	Left Cuneus	1.2 ± 0.2	1.1 ± 0.2	− 9	− 83	35	1909	** < 0.001**
4	631	Left Angular	1.4 ± 0.2	1.3 ± 0.2	− 45	− 74	32	1784	** < 0.001**
5	160	Right Superior Frontal	0.9 ± 0.1	0.8 ± 0.1	20	69	8	1753	** < 0.001**
6	360	Left Precentral	1.4 ± 0.2	1.3 ± 0.2	− 44	− 2	45	1744	** < 0.001**
7	13	Right Cerebellum	2.4 ± 0.3	2.3 ± 0.3	14	− 63	− 15	1721	** < 0.001**
8	273	Right Superior Medial Frontal	1.3 ± 0.2	1.2 ± 0.2	11	56	24	1720	** < 0.001**
9	46	Left Occipital Superior	0.9 ± 0.2	0.8 ± 0.2	− 14	− 99	12	1717	** < 0.001**
10	58	Right Medioorbital Frontal	0.9 ± 0.1	0.8 ± 0.1	9	69	− 8	1704	** < 0.001**
11	18	Left Occipital Superior	1.4 ± 0.2	1.3 ± 0.2	− 14	− 71	39	1684	** < 0.001**

The anatomical locations were based on the Automated Anatomical Labeling version 3 Atlas (AAL3). TFCE *p*-value < 0.05 family-wise error corrected, cluster-size threshold > 10 voxels; x, y, z = the original SPM coordinates in millimeters of the MNI space; in case of multiple peaks in the same anatomic area of a cluster, only the maximal peak is reported

Adjusted from age, sex, educational level, TIV, WMHs and MRI type

*CB* Counting backward, *NA* Naming animals

The study found that individuals with a high cost for naming animals (NA DTC) while performing a dual-task had lower GMV compared to those with a low NA DTC in 11 clusters. These clusters were in the left hemisphere in the lingual, cuneus, angular, precentral, and superior occipital gyri, and in the right hemisphere in the inferior temporal, superior and medio-orbital frontal gyri, and cerebellum (Table 2 and Fig. 2).

Additionally, Fig. 2 shows clusters (in yellow) that overlap in both dual-task conditions, located in the temporal lobe bilaterally.

There was no difference in WMHs volume according to the load of the DTC (CB and NA condition).

### Moderation analysis

Three models of moderation analysis were conducted to investigate whether the cognitive status of the subjects moderated the association between the DTC and GMV clusters.

The relationship between the CB DTC and GMV clusters was not found to be moderated by differences between the cognitive groups (including dementia vs. control groups, MCI vs. control groups, dementia vs. MCI groups).

**Fig. 3:**
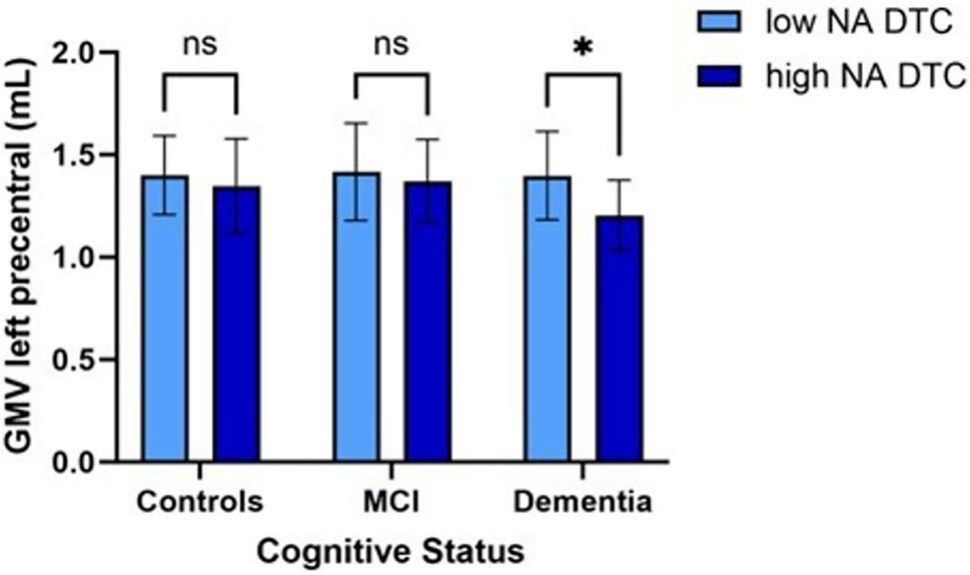
Gray Matter Volume concentration (GMV) in the left precentral gyrus according to the load of Naming Animal Dual-Task Cost (NA DTC) and the cognitive status: Low NA DTC in light blue and High NA DTC in dark blue in older adults with dementia, mild cognitive impairment (MCI) and control group

However, the association between NA DTC and GMV was moderated by cognitive status, with significant differences observed between subjects with dementia (overall: b = − 0.07, *p* = 0.03) versus controls subjects in a cluster located in the left precentral gyrus (Table 3 and Fig. 3). In the dementia group, lower GMV in the left precentral gyrus was associated with high NA DTC (conditional effect, b = − 0.18, *p* = 0.002), while this relationship was not significant in the control group (conditional effect, b = − 0.03, p = 0.47).

**Table 3:** Interaction Between the Naming Animal Dual-task Cost (NA DTC) Level and the Cognitive Status on the Gray Matter Volume (GMV) with a conditional effect when applicable

Predictor (GMv Cluster)	Moderator (Condition)	B	SE	*p-*value	95% CI
Left Precentral	**MCI versus Control groups (n = 289)**	0.01	0.05	0.85	(− 0.09, 0.11)
	**Dementia versus Control groups (n = 165)**	− 0.07	0.03	**0.03**	(− 0.14, − 0.01)
	Dementia group (n = 44)	− 0.18	0.06	**0.002**	(− 0.29, 0.07)
	Control group (n = 121)	− 0.03	0.04	0.47	(− 0.10, 0.05)
	**Dementia versus MCI (n = 212)**	− 0.17	0.06	**0.01**	(− 0.29, − 0.04)
	Dementia group (n = 44)	− 0.18	0.06	**0.002**	(− 0.29, − 0.07)
	MCI group (n = 168)	− 0.01	0.03	0.68	(− 0.07, 0.05)

For clarity, only significant results are shown in the table; for exhaustive results, see supplementary material

*P*-value significant (i.e., < 0.05) in bold

*MCI* Mild cognitive impairment

Additionally, the cognitive status comparing people with dementia versus MCI group significantly moderated the association between NA DTC and GMV loss in the left precentral gyrus (overall: b = − 0.17, *p* = 0.01, conditional effect for dementia group: b = − 0.18, *p* = 0.002 versus for MCI group conditional effect: b = − 0.01,* p* = 0.68) (Table 3 and Fig. 3).

No other moderation effect was found between groups (i.e. dementia group vs. control group nor MCI vs. control group nor MCI vs. dementia group) in the other clusters tested (Table 3 and Fig. 3).

## Discussion

This study aims to explore the neural substrate of high DTC across the cognitive spectrum. We found that older adults with a high DTC had smaller GMV in the bilateral middle and inferior temporal gyri. Furthermore, the moderation analysis indicated that individuals with dementia demonstrated an additional cluster where high NA DTC was linked to lower GMV in the left precentral gyrus, in comparison to the other cognitive groups, including the MCI and control groups.

In the entire cohort of older adults, poorer dual-task performance is significantly associated with smaller GMV in the anterior part of the lateral temporal lobes (middle and inferior gyri). The anterior part of the middle and inferior temporal gyri play a critical role in several cognitive processes, including semantic visual recognition, verbal semantic memory and language (Herlin et al. 2021). They are also essential for integrating sensory information and facilitating complex motor control (Rosso et al. 2013). Smaller GMV in these areas may indicate difficulties to handle the cognitive task while maintaining motor control, resulting in a high DTC.

The high CB DTC group was smaller than the high NA DTC group. It is noteworthy that the majority of individuals with elevated CB DTC scores were also included in the high NA DTC group. These findings indicate that individuals who experience difficulties with CB DTC may also encounter challenges with NA. Conversely, this is not necessarily the case, as there is a greater range of cognitive resources engaged by NA compared to CB, involving additional cognitive abilities beyond a simple backward counting by ones task (Beauchet et al. 2005).

Our study identified that NA DTC can be an index of extensive GMV loss in key brain areas related to motor and high-level cognitive dysfunctions, specifically in more advanced stages of cognitive decline. This is consistent with the naming animals task having a broader cortical demand. The smaller GMV in the right superior frontal and medio-orbito frontal gyri associated with high NA DTC, which play a role in executive functions, illustrates the known contribution of this region to dual-task gait alteration (Al-Yahya et al. 2011). The loss of GMV in the cerebellum associated with high NA DTC also plays a role in the coordination of complex motor control. These findings are consistent with those reported in previous studies, which have demonstrated that the cognitive-motor interaction involves specific functions, including attention, executive function, and coordination (Yogev-Seligmann et al. 2008; Al-Yahya et al. 2011). Additionally, they provide evidence regarding the neural substrate associated with this phenomenon.

In individuals with dementia, the study found that the left precentral gyrus, also known as the primary motor cortex (M1) is linked to high NA DTC, in contrast with other cognitive groups. This suggests that in people with dementia, the GMV loss in the M1, the region that initially serves as an effector of central motor command, is partially responsible for the observed impairment in dual-task gait. This supplementary affected region in the dementia group may elucidate why those individuals are more prone to experience high DTC as a consequence of the damage to neuronal integrity in a crucial area implicated in the central motor control. Therefore, individuals with dementia must manage their remaining intact brain resources and prioritize between motor and cognitive tasks for action execution, which frequently conduct to poorer dual-task performance.

In individuals with MCI, the neural substrate of high NA DTC is identical to that observed in the control group, affecting all aforementioned areas with the exception of the left precentral gyrus. However, individuals with MCI have higher clinical dual-task gait and cognitive impairments in comparison to the control group. This can be explained by the temporal sequence of neurodegeneration, whereby alterations in clinical gait performance precede structural alterations to the brain (Tian et al. 2023). This illustrates the interest of DTC as a clinical early marker of cognitive decline.

In contrast to the findings of other studies, our results did not indicate an association between CB DTC and the volume of WMHs. However, the high NA DTC group exhibited a higher volume of WMHs than the low NA DTC group. This association remains controversial in the literature (Subotic et al. 2023; Nadkarni et al. 2012; Blumen et al. 2023). Moreover, the method used in the current study to quantify these parameters was not specific enough because it only encompassed the whole brain, while associations were reported in specific regions of interest, such as the frontotemporal region. Additionally, the T1 was not as specific as a FLAIR and may have underestimated the volume of WMHs (Gaser et al. 2022). Furthermore, some authors attribute the burden of vascular abnormalities to be more linked to the motoric cognitive risk syndrome, which is another subgroup at high risk of dementia progression (Gomez et al. 2022). However, this subgroup was not considered in the current analysis.

This study contributes to the understanding of the underlying pathophysiological mechanisms of altered cognitive-motor interaction across the spectrum of cognitive decline in older adults, supported by a large sample size and a well-characterized population. However, several limitations must also be considered. First, the cross-sectional design does not permit causal conclusions. Second, all subjects were recruited from a memory clinic and some participants in the control group had subjective cognitive complaints. The dementia group had a lower number of participants than the others and comprised only nine individuals without AD. Furthermore, the inclusion of MMSE and/or ADAS-Cog as covariates in the VBM analysis resulted in a loss of statistical significance. It is probable that this is due to the correlation between DTC (both CB and NA) and these neuropsychological scores. The loss of significance after adjustment for global cognitive performance indicates that cognitive status plays a crucial role in the observational relationship between DTC and GMV. Additionally, the Montreal Cognitive Assessment (MoCA) could be used, as it has been shown to be more sensitive than the MMSE in detecting pre-dementia stages. Moreover, other gait parameters such as gait variability, (Pieruccini‐Faria et al. 2021) as well as combined marker like genomic analysis (Sakurai et al. 2024) should be considered for further analysis. Analyzing the cognitive load by accounting for the number of errors during the cognitive task would also be insightful (Goh et al. 2021). Finally, the use of two different field strengths in MRI (1.5 and 3 T), although adjusted for as a covariate, is another limitation. Nevertheless, the processing software used has been shown to be reliable across scanners and, the robustness of automated methods for brain volume measurement between 1.5 and 3 Tesla scanners has been previously established (Gaser et al. 2022; Heinen et al. 2016).

## Conclusions

The present study found an association between high DTC and reduced gray matter volume in bilateral temporal, left parieto-occipital, and right frontal areas in a cohort of older adults. Importantly, the smaller GMV in the left M1 associated with high DTC are only found in the dementia group and may be explain why these subjects experience more frequently dual-task gait disturbances. These findings contribute to the existing body of knowledge regarding the neural substrate of high DTC, a parameter that links cognitive and motor deficits in older adults. Future research directions may involve conducting longitudinal studies to validate these cross-sectional associations.

## Supplementary Information

Below is the link to the electronic supplementary material.

Supplementary file 1 (DOCX 25 KB)

## Data Availability

Data available upon reasonable request.

## References

[CR1] Allali G, Montembeault M, Saj A, Wong CH, Cooper-Brown LA, Bherer L, Beauchet O (2019) Structural brain volume covariance associated with gait speed in patients with amnestic and non-amnestic mild cognitive impairment: a double dissociation. Édité par Manuel Montero-Odasso et George Perry. J Alzheimer’s Disease 71(s1):S29-39. 10.3233/JAD-190038 31127784 10.3233/JAD-190038

[CR2] Al-Yahya E, Dawes H, Smith L, Dennis A, Howells K, Cockburn J (2011) Cognitive motor interference while walking: a systematic review and meta-analysis. Neurosci Biobehav Rev 35(3):715–728. 10.1016/j.neubiorev.2010.08.008 20833198 10.1016/j.neubiorev.2010.08.008

[CR3] Arlt S, Buchert R, Spies L, Eichenlaub M, Lehmbeck JT, Jahn H (2013) Association between fully automated mri-based volumetry of different brain regions and neuropsychological test performance in patients with amnestic mild cognitive impairment and Alzheimer’s disease. Eur Arch Psychiatry Clin Neurosci 263(4):335–344. 10.1007/s00406-012-0350-7 22940716 10.1007/s00406-012-0350-7

[CR4] Ashburner J, Friston KJ (2000) Voxel-based morphometry—the methods. NeuroImage 11(6): 805–821. 10.1006/nimg.2000.0582. 2011. Diffeomorphic Registration Using Geodesic Shooting and Gauss–Newton Optimisation. NeuroImage 55(3): 954–967. 10.1016/j.neuroimage.2010.12.049 10.1016/j.neuroimage.2010.12.049 PMC3221052 21216294

[CR5] Beauchet O, Dubost V, Gonthier R, Kressig RW (2005) Dual-task-related gait changes in transitionally frail older adults: the type of the walking-associated cognitive task matters. Gerontology 51(1):48–52. 10.1159/000081435 15591756 10.1159/000081435

[CR6] Beauchet O, Launay CP, Sekhon H, Montembeault M, Allali G (2019) Association of hippocampal volume with gait variability in Pre-dementia and dementia stages of alzheimer disease: results from a cross-sectional study. Exp Gerontol 115(janvier):55–61. 10.1016/j.exger.2018.11.010 30447261 10.1016/j.exger.2018.11.010

[CR7] Blumen HM, Jayakody O, Verghese J (2023) Gait in cerebral small vessel disease, Pre-Dementia, and Dementia: a systematic review. Int J Stroke off Je Int Stroke Soc 18(1):53–61. 10.1177/17474930221114562 10.1177/17474930221114562 PMC9841467 35797006

[CR8] Cullen S, Borrie M, Carroll S, Sarquis-Adamson Y, Pieruccini-Faria F, McKay S, Montero-Odasso M (2019) Are Cognitive Subtypes Associated with Dual-Task Gait Performance in a Clinical Setting? Édité par Manuel Montero-Odasso et George Perry. J Alzheimer’s Disease 71(s1):S57-64. 10.3233/JAD-181196 31322559 10.3233/JAD-181196

[CR9] Dion M, Potvin O, Belleville S, Ferland G, Renaud M, Bherer L, Joubert S et al (2015) Normative Data for the Rappel libre/Rappel indicé à 16 items (16-item Free and Cued Recall) in the elderly quebec-french population. Clin Neuropsychol 28(sup1):1–19. 10.1080/13854046.2014.915058 10.1080/13854046.2014.915058 24815338

[CR10] Doi T, Blumen HM, Verghese J, Shimada H, Makizako H, Tsutsumimoto K, Hotta R, Nakakubo S, Suzuki T (2017) Gray Matter Volume and Dual-Task Gait Performance in Mild Cognitive Impairment. Brain Imaging Behav 11(3):887–898. 10.1007/s11682-016-9562-1 27392792 10.1007/s11682-016-9562-1 PMC5266675

[CR11] Dubois B, Slachevsky A, Litvan I, Pillon B (2000) The FAB: a frontal assessment battery at bedside. Neurology 55(11):1621–1626. 10.1212/wnl.55.11.1621 11113214 10.1212/wnl.55.11.1621

[CR12] Dubois B, Feldman HH, Jacova C, Dekosky ST, Barberger-Gateau P, Cummings J, Delacourte A et al (2007) Research Criteria for the Diagnosis of Alzheimer’s Disease: Revising the NINCDS-ADRDA Criteria. Lancet Neurol 6(8):734–746. 10.1016/S1474-4422(07)70178-3 17616482 10.1016/S1474-4422(07)70178-3

[CR13] European GAITRite® Network Group, Reto W. Kressig, et Olivier Beauchet (2006) Guidelines for Clinical Applications of Spatio-Temporal Gait Analysis in Older Adults. Ag Clin Exp Res18(2):174–176. 10.1007/BF03327437. 10.1007/BF03327437 16702791

[CR14] Folstein MF, Folstein SE, McHugh PR (1975) “Mini-Mental State” A Practical Method for Grading the Cognitive State of Patients for the Clinician. J Psychiatric Res 12(3):189–198. 10.1016/0022-3956(75)90026-6 10.1016/0022-3956(75)90026-6 1202204

[CR15] Gaser C, Dahnke R, Thompson PM, Kurth F, Luders E, Alzheimer’s Disease Neuroimaging Initiative (2022) CAT–A Computational Anatomy Toolbox for the Analysis of Structural MRI Data. Preprint. Neuroscience. 10.1101/2022.06.11.495736

[CR16] Goh H-T, Pearce M, Vas A (2021) Task matters: an investigation on the effect of different secondary tasks on dual-task gait in older adults. BMC Geriatr 21(1):510. 10.1186/s12877-021-02464-8 34563129 10.1186/s12877-021-02464-8 PMC8465774

[CR17] Gomez GT, Gottesman RF, Gabriel KP, Palta P, Gross AL, Soldan A, Albert MS et al (2022) The association of motoric cognitive risk with incident dementia and neuroimaging characteristics: the atherosclerosis risk in communities study. Alzheimer’s Dementia 18(3):434–444. 10.1002/alz.12412 34786837 10.1002/alz.12412 PMC10064850

[CR18] Heinen R, Bouvy WH, Mendrik AM, Viergever MA, Biessels GJ, de Bresser J (2016) Robustness of automated methods for brain volume measurements across different MRI Field Strengths. PLoS ONE 11(10):e0165719. 10.1371/journal.pone.0165719 27798694 10.1371/journal.pone.0165719 PMC5087903

[CR19] Herlin B, Navarro V, Dupont S (2021) The temporal pole: from anatomy to function—a literature appraisal. J Chem Neuroanatomy 113(avril):101925. 10.1016/j.jchemneu.2021.101925 10.1016/j.jchemneu.2021.101925 33582250

[CR20] Hupfeld KE, Geraghty JM, McGregor HR, Hass CJ, Pasternak O, Seidler RD (2022) Differential relationships between brain structure and dual task walking in young and older adults. Front Ag Neurosci 14(mars):809281. 10.3389/fnagi.2022.809281 10.3389/fnagi.2022.809281 PMC8963788 35360214

[CR21] Karas GB, Scheltens P, Rombouts SA, Visser PJ, van Schijndel RA, Fox NC, Barkhof F (2004) Global and Local Gray Matter Loss in Mild Cognitive Impairment and Alzheimer’s Disease. Neuroimage 23(2):708–716. 10.1016/j.neuroimage.2004.07.006 15488420 10.1016/j.neuroimage.2004.07.006

[CR22] Koppelmans V, Silvester B, Duff K (2022) Neural mechanisms of motor dysfunction in mild cognitive impairment and Alzheimer’s disease: a systematic review. J Alzheimer’s Dis Reports 6(1):307–344. 10.3233/ADR-210065 10.3233/ADR-210065 PMC9277676 35891638

[CR23] Kueper JK, Speechley M, Montero-Odasso M (2018) The Alzheimer’s Disease Assessment Scale-Cognitive Subscale (ADAS-Cog): modifications and Responsiveness in Pre-Dementia Populations. A Narrative Review. J Alzheimer’s Dis 63(2):423–444. 10.3233/JAD-170991 29660938 10.3233/JAD-170991 PMC5929311

[CR24] Montero-Odasso M, Bergman H, Phillips NA, Wong CH, Sourial N, Chertkow H (2009a) Dual-Tasking and Gait in People with Mild Cognitive Impairment. The Effect of Working Memory. BMC Geriatr 9:41. 10.1186/1471-2318-9-41 19723315 10.1186/1471-2318-9-41 PMC2748075

[CR25] Montero-Odasso M, Casas A, Hansen KT, Bilski P, Gutmanis I, Wells JL, Borrie MJ (2009b) Quantitative gait analysis under dual-task in older people with mild cognitive impairment: a reliability study. J Neuroeng Rehabil 6(1):35. 10.1186/1743-0003-6-35 19772593 10.1186/1743-0003-6-35 PMC2754991

[CR26] Montero-Odasso MM, Sarquis-Adamson Y, Speechley M, Borrie MJ, Hachinski VC, Wells J, Riccio PM et al (2017) Association of Dual-Task gait with incident dementia in mild cognitive impairment: results from the gait and brain study. JAMA Neurol 74(7):857. 10.1001/jamaneurol.2017.0643 28505243 10.1001/jamaneurol.2017.0643 PMC5710533

[CR27] Montero-Odasso M, Almeida QJ, Bherer L, Burhan AM, Camicioli R, Doyon J, Fraser S et al (2019) Consensus on shared measures of mobility and cognition: from the Canadian consortium on neurodegeneration in aging (CCNA). J Gerontol Ser A 74(6):897–909. 10.1093/gerona/gly148 10.1093/gerona/gly148 PMC6521916 30101279

[CR28] Muir SW, Speechley M, Wells J, Borrie M, Gopaul K, Montero-Odasso M (2012) Gait assessment in mild cognitive impairment and Alzheimer’s Disease: the effect of dual-task challenges across the cognitive spectrum. Gait Posture 35(1):96–100. 10.1016/j.gaitpost.2011.08.014 21940172 10.1016/j.gaitpost.2011.08.014

[CR29] Nadkarni NK, Levine B, McIlroy WE, Black SE (2012) Impact of Subcortical Hyperintensities on Dual-Tasking in Alzheimer Disease and Aging. Alzheimer Dis Assoc Disord 26(1):28–35. 10.1097/WAD.0b013e3182172c58 21502852 10.1097/WAD.0b013e3182172c58 PMC3874593

[CR30] Pennanen C, Testa C, Laakso M, Hallikainen M, Helkala E, Hanninen T, Kivipelto M et al (2005) A voxel based morphometry study on mild cognitive impairment. J Neurol Neurosurg Psychiatry 76(1):11–14. 10.1136/jnnp.2004.035600 15607988 10.1136/jnnp.2004.035600 PMC1739300

[CR31] Pieruccini-Faria F, Black SE, Masellis M, Smith EE, Almeida QJ, Li KZH, Bherer L, Camicioli R, Montero-Odasso M (2021) Gait variability across neurodegenerative and cognitive disorders: results from the Canadian consortium of neurodegeneration in aging (CCNA) and the Gait and Brain Study. Alzheimer’s Dementia 17(8):1317–1328. 10.1002/alz.12298 33590967 10.1002/alz.12298 PMC8451764

[CR32] Plummer P, Eskes G (2015) Measuring treatment effects on dual-task performance: a framework for research and clinical practice. Front Human Neurosci 9(avril):225. 10.3389/fnhum.2015.00225 10.3389/fnhum.2015.00225 PMC4412054 25972801

[CR33] Quinn BP (1999) Diagnostic and Statistical Manual of Mental Disorders, Fourth Edition, Primary Care Version. Primary Care Companion J Clin Psychiatry 1(2):54–55.

[CR34] Rolls ET, Huang CC, Lin CP, Feng J, Joliot M (2020) Automated Anatomical Labelling Atlas 3. Neuroimage 206(février):116189. 10.1016/j.neuroimage.2019.116189 31521825 10.1016/j.neuroimage.2019.116189

[CR35] Rosso AL, Studenski SA, Chen WG, Aizenstein HJ, Alexander NB, Bennett DA, Black SE et al (2013) Aging, the Central Nervous System, and Mobility. J Gerontol A Biol Sci Med Sci 68(11):1379–1386. 10.1093/gerona/glt089 23843270 10.1093/gerona/glt089 PMC3805295

[CR36] Sakurai R, Bartha R, Montero-Odasso M (2019) Entorhinal cortex volume is associated with Dual-Task Gait Cost Among Older Adults With MCI: results from the gait and brain study. J Gerontol Ser A 74(5):698–704. 10.1093/gerona/gly084 10.1093/gerona/gly084 PMC6477635 29767690

[CR37] Sakurai R, Pieruccini-Faria F, Cornish B, Fraser J, Binns MA, Beaton D, Dilliott AA, Kwan D, Ramirez J, Tan B, Scott CJ (2024) Link among Apolipoprotein E E4, Gait, and Cognition in Neurodegenerative Diseases: ONDRI Study. Alzheimer’s Dementia 15(avril):2968–2979. 10.1002/alz.13740 10.1002/alz.13740 PMC11032526 38470007

[CR38] Spisák T, Spisák Z, Zunhammer M, Bingel U, Smith S, Nichols T, Kincses T (2019) Probabilistic TFCE: a generalized combination of cluster size and voxel intensity to increase statistical power. Neuroimage 185(janvier):12–26. 10.1016/j.neuroimage.2018.09.078 30296561 10.1016/j.neuroimage.2018.09.078 PMC6834440

[CR39] Subotic A, Gee M, Nelles K, Ba F, Dadar M, Duchesne S, Sharma B et al (2023) Gray matter loss relates to dual task gait in lewy body disorders and aging. J Neurol. 10.1007/s00415-023-12052-y 37902878 10.1007/s00415-023-12052-y

[CR40] Tian Qu, Montero-Odasso M, Buchman AS, Mielke MM, Espinoza S, DeCarli CS, Newman AB et al (2023) Dual Cognitive and Mobility Impairments and Future Dementia-Setting a Research Agenda. Alzheimer’s Dementia J Alzheimer’s Asso 19(4):1579–1586. 10.1002/alz.12905 10.1002/alz.12905 PMC10101877 36637077

[CR41] Tripathi S, Verghese J, Blumen HM (2019) Gray Matter Volume Covariance Networks Associated with Dual-Task Cost during Walking-While-Talking. Hum Brain Mapp 40(7):2229–2240. 10.1002/hbm.24520 30664283 10.1002/hbm.24520 PMC6445705

[CR42] Warrington EK, James M (1991) The Visual Object and Space Perception Battery. Pearson, London

[CR43] Winblad B, Palmer K, Kivipelto M, Jelic V, Fratiglioni L, Wahlund L-O, Nordberg A et al (2004) Mild Cognitive Impairment–beyond Controversies, towards a Consensus: Report of the International Working Group on Mild Cognitive Impairment. J Intern Med 256(3):240–246. 10.1111/j.1365-2796.2004.01380.x 15324367 10.1111/j.1365-2796.2004.01380.x

[CR44] Yogev-Seligmann G, Hausdorff JM, Giladi N (2008) The Role of Executive Function and Attention in Gait. Movement Dis off J Movement Disorder Soc 23(3):329–342. 10.1002/mds.21720. (**quiz 472**) 10.1002/mds.21720 PMC2535903 18058946

