# Supplementary material for: Mapping the neural substrate of high dual-task gait cost in older adults across the cognitive spectrum
Source: Brain Struct Funct. 2025 Jan 4;230(1):25. doi: 10.1007/s00429-024-02873-6 (PMC11700055; doi:10.1007/s00429-024-02873-6)
Supplement: Supplementary file 1 — (DOCX 25 KB) [file 429_2024_2873_MOESM1_ESM.docx]

**Supplementary material**

eTable1: Demographic Characteristics of the Participants According to the Load of Dual Task Cost:

| **Total population** | **CB DTC <20%** | **CB DTC ≥20%** | *p* value |
| --- | --- | --- | --- |
| **(n = 336)** | n=294 | n=42 |  |
| Age (years), (mean±SD) | 72 ± 5.2 | 74 ± 6.7 | 0.093 |
| Women, n (%) | 123 (42%) | 23 (55%) | 0.118 |
| Post secondary education, n (%) | 170 (58%) | 18 (43%) | 0.064 |
| Body mass index (kg/cm^2^) (mean ±SD) | 26.1 ± 4.0 | 26.5 ± 4.2 | 0.515 |
| Number of comorbidities (mean±SD) | 2.4 C1.8 | 2.3 ± 1.9 | 0.745 |
| Cognitive Profile | Controls= 116 (95%) | 6 (5%) | **0.003** |
| n (%) | MCI=142 (84%) | 26 (16%) | **0.003** |
|  | Dementia= 36 (78%) | 10 (22%) | **0.003** |
| Total intracranial volume (mL) adjusted on sex, (mean ±SD) | 1503 ± 139 | 1492 ± 132 | 0.658 |
| Volume of white matter hyperintensities (mL) | 5.3 ± 7.5 | 6.2 ± 10.3 | 0.814 |
| MMSE score † (mean±SD) | 27.3 ± 2.6 | 26.4 ± 2.9 | **0.032** |
| ADAS-Cog score ‡ (mean±SD) | 5.8 ± 3.4 | 8.3 ± 7.2 | **<0.001** |

| **Total population** | **NA DTC <20%** | **NA DTC ≥20%** |  |
| --- | --- | --- | --- |
| **(n = 336)** | n=199 | n=134 | *p* value |
| Age (years), (mean±SD) | 72 ± 4.8 | 74 ± 6.0 | **0.030** |
| Women, n (%) | 77 (38%) | 67 (50%) | **0.041** |
| Post secondary education, n (%) | 119 (60%) | 70 (52%) | 0.172 |
| Body mass index (kg/cm^2^) (mean ±SD) | 26.3 ± 3.9 | 25.9 ± 4.2 | 0.45 |
| Number of comorbidities (mean±SD) | 2.3 ± 1.8 | 2.4 ± 1.7 | 0.514 |
| Cognitive Profile | Controls= 87 (44%) | 34 (25%) | **<0.001** |
| n (%) | MCI=94 (47%) | 74 (55%) | **<0.001** |
|  | Dementia=18 (9%) | 26 (19%) | **<0.001** |
| Total intracranial volume (mL) adjusted on sex, (mean ±SD) | 1515 ± 138 | 1484 ± 137 | **0.047** |
| Volume of white matter hyperintensities (mL) | 4.5 ± 6.2 | 6.4 ± 9.4 | **0.020** |
| MMSE score † (mean±SD) | 27.4 ± 2.4 | 26.8 ± 2.9 | 0.051 |
| ADAS-Cog score ‡ (mean±SD) | 5.5 ± 3.0 | 6.8 ± 5.2 | **0.009** |

*NOTE. Where appropriate the mean is shown with standard deviation in parentheses.*

*Comparison based on a two samples t-test or Chi-square test as appropriate.*

*P-value significant (i.e,<0.05) in bold.*

*1individual in the control group and 2 participants with dementia didn’t perform the naming animals task.*

*Abbreviations:*

*SD: standard deviation*

*n, number of participants*

† *MMSE= Folstein Mini-mental State Examination (scores range from 0 to 30, higher scores representing better function),*

‡ *ADAS-Cog= Alzheimer’s Disease Assessment Scale (score ranges from 0 to 70 with higher scores suggesting greater impairment, 58 missing data),*

eTable 2: Interaction Between the Naming Animal Dual-task Cost (NA DTC) Level and the Cognitive Status on the Gray Matter Volume (GMV):

| **Moderator: Dementia vs. Controls** | | | | | |
| --- | --- | --- | --- | --- | --- |
| **Cluster No.** | **Predictor** | **B** | **SE** | **P-value** | **95% CI** |
| C1 | Left lingual | 0.025 | 0.027 | 0.36 | -0.029 0.078 |
| C2 | Right inferior temporal | 0.004 | 0.032 | 0.90 | -0.059 0.067 |
| C3 | Left cuneus | -0.06 | 0.032 | 0.09 | -0.11 0.01 |
| C4 | Left angular | -0.66 | 0.034 | 0.06 | -0.13 0.002 |
| C5 | Right superior frontal | -0.001 | 0.02 | 0.99 | -0.04 0.04 |
| C6 | Left precentral | -0.07 | 0.03 | **0.03** | -0.14 -0.01 |
| C7 | Right cerebellum | -0.08 | 0.05 | 0.11 | -0.17 0.02 |
| C8 | Right superior medial frontal | -0.03 | 0.02 | 0.26 | -0.07 0.02 |
| C9 | Left occipital superior | -0.01 | 0.03 | 0.62 | -0.06 0.04 |
| C10 | Right medio-orbitofrontal | -0.001 | 0.02 | 0.97 | -0.03 0.03 |
| C11 | Left occipital superior | -0.03 | 0.04 | 0.32 | -0.11 0.04 |
| **MCI vs. Controls** | | | | | |
|  | **Predictor** | **B** | **SE** | **P-value** | **95% CI** |
| C1 | Left lingual | 0.04 | 0.04 | 0.29 | -0.04 0.12 |
| C2 | Right inferior temporal | -0.07 | 0.04 | 0.10 | -0.15 0.01 |
| C3 | Left cuneus | -0.02 | 0.05 | 0.69 | -0.12 0.08 |
| C4 | Left angular | -0.04 | 0.05 | 0.44 | -0.13 0.06 |
| C5 | Right superior frontal | -0.02 | 0.03 | 0.60 | -0.07 0.04 |
| C6 | Left precentral | 0.01 | 0.05 | 0.84 | -0.09 0.11 |
| C7 | Right cerebellum | -0.11 | 0.07 | 0.14 | -0.25 0.04 |
| C8 | Right superior medial frontal | -0.05 | 0.04 | 0.17 | -0.13 0.02 |
| C9 | Left occipital superior | 0.01 | 0.04 | 0.76 | -0.07 0.09 |
| C10 | Right medio-orbitofrontal | -0.03 | 0.02 | 0.24 | -0.08 0.02 |
| C11 | Left occipital superior | -0.01 | 0.05 | 0.91 | -0.11 0.10 |
| **Dementia vs. MCI** | | | | | |
|  | **Predictor** | **B** | **SE** | **P-value** | **95% CI** |
| C1 | Left lingual | 0.003 | 0.05 | 0.95 | -0.09 0.10 |
| C2 | Right inferior temporal | 0.07 | 0.06 | 0.25 | -0.05 0.19 |
| C3 | Left cuneus | -0.10 | 0.07 | 0.16 | -0.23 0.04 |
| C4 | Left angular | -0.10 | 0.07 | 0.16 | -0.24 0.04 |
| C5 | Right superior frontal | 0.002 | 0.04 | 0.95 | -0.08 0.08 |
| C6 | Left precentral | -0.17 | 0.06 | **0.01** | -0.29 -0.04 |
| C7 | Right cerebellum | -0.03 | 0.10 | 0.73 | -0.23 0.16 |
| C8 | Right superior medial frontal | -0.01 | 0.05 | 0.89 | -0.12 0.10 |
| C9 | Left occipital superior | -0.04 | 0.06 | 0.44 | -0.15 0.07 |
| C10 | Right medio-orbitofrontal | 0.02 | 0.04 | 0.60 | -0.05 0.09 |
| C11 | Left occipital superior | -0.06 | 0.06 | 0.37 | -0.20 0.07 |
